# Implementation of artificial intelligence (AI) applications in radiology: hindering and facilitating factors

**DOI:** 10.1007/s00330-020-06946-y

**Published:** 2020-05-26

**Authors:** Lea Strohm, Charisma Hehakaya, Erik R. Ranschaert, Wouter P. C. Boon, Ellen H. M. Moors

**Affiliations:** 1 Innovation Studies, Copernicus Institute of Sustainable Development, University Utrecht, Utrecht, The Netherlands; 2 Division of Imaging & Oncology, University Medical Center Utrecht, Utrecht, The Netherlands; 3 Department of Radiology, Elisabeth-TweeSteden Ziekenhuis, Tilburg, The Netherlands

**Keywords:** Artificial intelligence, Computer systems, Diagnosis, Computer-assisted, Information systems, Radiology

## Abstract

**Objective:**

The objective was to identify barriers and facilitators to the implementation of artificial intelligence (AI) applications in clinical radiology in The Netherlands.

**Materials and methods:**

Using an embedded multiple case study, an exploratory, qualitative research design was followed. Data collection consisted of 24 semi-structured interviews from seven Dutch hospitals. The analysis of barriers and facilitators was guided by the recently published Non-adoption, Abandonment, Scale-up, Spread, and Sustainability (NASSS) framework for new medical technologies in healthcare organizations.

**Results:**

Among the most important facilitating factors for implementation were the following: (i) pressure for cost containment in the Dutch healthcare system, (ii) high expectations of AI’s potential added value, (iii) presence of hospital-wide innovation strategies, and (iv) presence of a “local champion.” Among the most prominent hindering factors were the following: (i) inconsistent technical performance of AI applications, (ii) unstructured implementation processes, (iii) uncertain added value for clinical practice of AI applications, and (iv) large variance in acceptance and trust of direct (the radiologists) and indirect (the referring clinicians) adopters.

**Conclusion:**

In order for AI applications to contribute to the improvement of the quality and efficiency of clinical radiology, implementation processes need to be carried out in a structured manner, thereby providing evidence on the clinical added value of AI applications.

**Key Points:**

• *Successful implementation of AI in radiology requires collaboration between radiologists and referring clinicians.*

• *Implementation of AI in radiology is facilitated by the presence of a local champion.*

• *Evidence on the clinical added value of AI in radiology is needed for successful implementation.*

**Electronic supplementary material:**

The online version of this article (10.1007/s00330-020-06946-y) contains supplementary material, which is available to authorized users.

## Introduction

Artificial intelligence (AI) is increasingly being recognized as an important application in clinical radiology [1–5]. Recent advances in machine learning have produced algorithms that allow automated and accurate detection and diagnosis of medical images. The large technological improvements have created high expectations among radiologists, healthcare providers, and policymakers alike. They promise considerable efficiency and quality gains for healthcare, for example by allowing more precise diagnosis and automating labor-intensive tasks, currently performed by radiologists [3, 6].

AI is expected to cause large changes in clinical work practices and requires complementary skills from radiologists [5, 7, 8]. A narrative of “radiologists becoming replaced by AI” has emerged, as discussions about this topic have flooded major conferences and scientific publications [8, 9]. Unsurprisingly, the replacement narrative has triggered strong responses within the radiology profession [1, 3, 10, 11]. While technical performance of AI applications is expected to increase continuously, its implementation in clinical radiology practice is rather complex and has so far been slow [6, 12, 13]. Earlier forms of AI applications, such as the first computerized aided diagnosis (CAD) systems, have failed to achieve widespread adoption. Literature has mainly blamed this on low technical performance of these early applications [5, 14–16], while other potential barriers to successful implementation, such as organizational or social aspects, have been largely ignored [17].

Technology implementation in hospital settings involves a large variety of stakeholders and organizational procedures, with strong routines and professional identities, as well as strict legal and regulatory standards [18–20]. Considering that AI applications in radiology are in an emerging phase, it is too early to evaluate their implementation [21]. However, in view of the unsuccessful widespread diffusion of earlier CAD systems, it can be assumed that AI applications will encounter barriers to implementation. We studied the facilitating and hindering factors for the successful implementation of AI applications in radiology departments, including not only technological but also organizational and social aspects.

## Materials and methods

In this study, we use the Non-adoption, Abandonment, Scale-up, Spread, and Sustainability (NASSS) framework [20] to identify the success and failure factors in the implementation of AI applications in clinical radiology, thereby also focusing on the socio-organizational aspects. The NASSS framework aims to detect the determinants of implementation processes of complex technologies in healthcare on seven domains: the condition, technology, value proposition, the adopter system (patient, lay caregivers, individual technology user, and other staff), the organization, and the wider institutional and social context. The framework takes a dynamic perspective by following the interactions between these domains over time. Based on this framework, we created propositions on potential facilitating and hindering factors.

We used an embedded multiple case study approach, investigating seven Dutch hospitals. Appendix A provides an overview of our interviewees and the cases, i.e. hospitals, they are involved in. We chose to focus on The Netherlands given the high pressure for cost savings in the health care sector, to which AI in radiology is expected to contribute significantly [22–24]. The Dutch radiology departments vary strongly in the number and types of internally available AI applications. These range from the detection and quantification of lung nodules in CT scans, mammography CAD systems, to stroke detection and automated bone-age assessment. We focused on those departments that used BoneXpert, a software-only medical device commercially distributed by Visiana since 2009. It runs automated bone maturity assessments based on X-rays of pediatric patients’ hands. BoneXpert is one of the first commercial applications of AI in radiology [25] and appeared to be the only AI application in clinical use across several hospitals in The Netherlands.^1^

Using a maximum variability logic, interviewees occupying different positions within the participating hospitals were selected (see Table 1 and Appendix A). Interviewees were contacted based on their experience with BoneXpert specifically or the implementation of AI applications for radiology more generally, as indicated by publicly available information or internal referral. The number of interviewees varied from one to four participants per hospital. Furthermore, four key informant interviews were used to investigate the external context of the cases, such as socio-economic and regulatory influences.

**Table 1:** Overview cases and interviewees

	Number of interviews	Roles of interviewees
Cases (7 hospitals)		
TKZ1	4	Senior radiologist; legal consultant; clinical physicist; operational department manager
TKZ2	4	Senior radiologist (2); junior technical physician; innovation manager
UMC1	4	Senior radiologists (3); innovation manager
UMC2	3	Junior radiologist (2); senior data scientist,
UMC3	3	Senior radiologist (2); senior data scientist
UMC4	1	Senior radiologist
AZ1	1	Senior radiologist
Key informants		
Professional organization	1	Member of management
Professional organization	1	Implementation advisor
Professional organization	1	Member of management
Imaging technology provider	1	Innovation manager
Total number of interviews	24	

From February to June 2019, 24 semi-structured interviews were conducted by a single researcher, complemented by a document analysis based on internal documents from the respective cases and publicly-available documents. Interviews were conducted until thematic saturation was reached, meaning when no new themes appeared during additional interviews. Twenty-one interviews were conducted face-to-face; three were held by telephone. Interviews were held in English if possible and lasted between 20 and 80 min. Oral permission for recording was granted by all interviewees. The interviews were subsequently transcribed and coded in NVivo. The concepts identified in the interviews were compared with the original NASSS framework, which was afterwards refined.

## Results

We first present the facilitating factors, followed by the hindering factors for AI implementation in radiology.

### Identified facilitating factors for AI implementation in radiology

Table 2 provides an overview of the number and identity of interviewees referencing the facilitating factors.

**Table 2:** Overview of facilitating factors for AI implementation

Facilitating factors	Interviewees (by interviewee ID, following Appendix A)	Sum
Pressure on healthcare budgets	4, 20, 19, 18, 22	5
Expected added benefit: improved diagnostic practice	1, 2, 3, 4, 5, 6, 7, 8, 10, 11, 12, 13, 14, 16, 17, 20, 22, 23	18
Expected added benefit: operational benefits	1, 2, 3, 4, 5, 6, 7, 8, 10, 11, 12, 13, 14, 16, 20, 22, 23	17
Easy integration in PACS	2, 3, 5, 6, 7, 8, 9, 10, 11, 12, 13,14, 16, 20, 22	15
Minimize workflow changes	1, 2, 4, 5, 6, 7, 8, 10, 11, 12, 17, 14, 20, 22	14
BoneXpert smooth integration in PACS	1, 3, 5, 7, 8, 9, 10, 13, 14, 22	10
Innovation strategy		4/7 hospitals
Innovation manager		3/7 hospitals
Local champions	1, 2, 3, 8, 10, 12, 14, 17, 22, 23,	10

First, *pressure on healthcare budgets* stimulates Dutch hospitals and radiology departments to develop and implement innovative technologies that promise efficiency and/or quality gains. The Dutch healthcare system is confronted with a constant rise in demand accompanied by a strong pressure to limit associated costs [27]. This context creates a favorable political context for AI applications.

Second, although there is little empirical evidence, radiologists, members of hospital management, and technology developers *expect a large added value* of the AI applications in clinical practice. The interviewees mentioned two main benefits: (1) improved diagnostic practice due to more precise and objective diagnoses, avoidance of mistakes, and the automation of cumbersome tasks; and (2) operational benefits, such as diminishing workloads, time-saving, more consistent reporting across radiologists, and advanced service availability.

Third, in order for AI applications to be perceived as user-friendly by radiologists, they need to be *easily integrated into existing IT systems* used by radiologists, such as picture archiving and communication systems (PACSs). This means that the output of the AI application should be displayed with the least possible clicks. Also, AI applications should be implemented without large changes to routines and workflow practices, i.e., by avoiding additional steps for reporting the result of the AI application. For example, the interviewed users experienced the integration of BoneXpert into the PACS as very smooth, being the main reason for its perceived user-friendliness. However, concerns remain about the integration of other AI applications into the PACSs.

Fourth, the openness towards AI application in radiology is expressed by the adoption of hospital-wide or radiology department–specific *innovation strategies*. In four of the seven cases, a hospital-wide innovation strategy including AI was present, reflecting innovation leadership among the hospital management. Such leadership is also manifested by the presence or absence of a designated innovation manager (present in three of the seven cases). On radiology department-level, only one hospital had a formalized innovation strategy regarding AI. However, four more hospitals were developing such a strategic approach at the time of this research.

Fifth, interviewees and document analysis [28, 29] show that *local champions* are vital in initiating and stimulating implementation within their department and taking the lead during the entire process. These local champions are radiologists that show a particularly strong interest in AI applications and usually have better than average understanding of the technical aspects of AI applications. To overcome the opposition of potential skeptical colleagues, local champions appear to follow two strategies: (1) providing general information on AI or on an AI application in particular through scientific articles, books, and presentations; and (2) promoting opportunities for experimentation with an application, e.g., by organizing showcases or installing a test version of the application. Both strategies aim to build trust and serve to familiarize and convince other radiologists (direct adopters) and referring clinicians (indirect adopters) with the AI application.

Finally, the Radiological Society of The Netherlands (NVvR) serves as a *knowledge-exchange platform* among its members facilitating the implementation of AI applications. The NVvR has included AI in its strategic research agenda since 2017 [28] and has a “technology committee,” i.e., a study group that raises awareness among Dutch radiologists, e.g., by organizing regular open meetings, advising hospitals on the development of an AI strategy, and pursuing the inclusion of AI in the curriculum for future radiologists.

### Identified hindering factors for AI implementation in radiology

Table 3 provides an overview of the number and identity of interviewees referencing the hindering factors. First, users perceived the technical performance of most AI applications as inconsistent. Technically, this refers to the algorithms’ performance, i.e., the sensitivity (number of false positives) and specificity (number of false negatives). In clinical terms, a large number of false positives create additional work for the radiologist, which was the case of earlier unsuccessful CAD applications. Having a large number of false negatives is even more dangerous, because it means that a potential lesion might get overlooked. Against a background of lacking technical understanding of AI, some radiologists are doubting the quality and safety of an application and fail to adopt or abandon AI applications. The interviews showed that computer science and programming knowledge required in the development of AI algorithms are not present-day competencies of radiologists. However, some technical understanding is imperative for quality and safety assessment and therefore create trust in the AI application’s reliability [8, 21, 30].

**Table 3:** Overview of hindering factors for AI implementation

Hindering factors	Interviewees (by interviewee ID, following Appendix A)	Sum
Inconsistent technical performance	3, 5, 6, 10, 11, 12, 13, 14, 22, 23	10
Doubting quality and safety of the application	2, 3, 4, 5, 6, 10, 11, 12, 13, 14	10
Technical knowledge necessary	1, 2, 3, 5, 8, 11, 12, 13, 14, 20	10
Unstructured planning and monitoring	2, 3, 5, 8, 9, 12, 14, 16, 17, 22	10
Unstructured implementation in workflow	3, 4, 5, 7, 9, 11, 12, 13, 16,	9
Absence of guidelines/best practices	3, 4, 5, 9, 12, 15, 16, 19	8
No empirical evidence on AI apps (validation)	3, 4, 5, 8, 9, 12, 13, 20, 23	9
Uncertain funding	1, 2, 3, 4, 6, 7, 8, 11, 12, 13, 14, 16, 18, 22	14
Limited communication between departments	6, 7, 9, 10, 17, 19, 20, 22	8
Inconsistent acceptance/trust of radiologists	1, 2, 3, 5, 6, 7, 8, 9, 10, 11, 12, 13, 14, 22	14
Acceptance trust of referring clinicians	1, 2, 4, 7, 10, 22	6
Inconsistent Acceptance of BoneXpert	1, 3, 5, 7, 8, 9, 10, 13, 14, 22	10
Reframe professional identity/responsibilities	2, 3, 4, 5, 6, 7, 13, 22	8
Framing/narrative as co-pilot	2, 3, 12, 14, 16	5
Regulatory and legal uncertainties	3, 4, 8, 11, 13, 15, 17, 23	8
Reference to post-market surveillance MDR	3, 4, 19, 20, 22	5
Legal responsibility for mistakes	4, 8, 11, 15, 17	5

Second, *planning and monitoring* of AI implementation tend to be unstructured. From an organizational perspective, clinical benefits or organizational goals that might be achieved by using AI applications are not clearly established ex-ante and therefore hard to assess after implementation. From a workflow perspective, implementation plans do not specify how the AI application should be integrated into the clinical workflow, which leads to significant variations in the way the application is used in different departments. Furthermore, in all cases, the work done to monitor existing practices or the impact of the implementation of novel technologies on the level of the hospital is currently limited. The unstructured nature of implementation processes can be explained by the absence of official guidelines or best practices.

Third, there is *a lack of empirical evidence* on the effect of AI applications on the radiological workflow, as well as their added value for clinical radiology practice. One reason is that measuring clinical and organizational benefits of AI on a micro-level is difficult. There is, for example, no standard methodology to measure increases in the quality of diagnosis. When evidence on the technical performance is available (such as with BoneXpert), interviewees noted that publications on the validation of the algorithms are based on laboratory rather than clinical settings.

Fourth, *funding* for AI applications is uncertain due to the lacking evidence on the added value of AI applications needed to back adoption and funding decisions. Moreover, the benefits and costs of using AI may be unequally divided over departments, which complicates funding decisions. If other departments are to cover parts of the additional costs, they need to learn about the technology and the potential benefits of using AI applications. This requires efficient communication between departments, a shortcoming in several of the studied cases.

Fifth, the *acceptance and trust* of direct (radiologists) and indirect adopters (referring clinicians) in AI applications differ greatly. Radiologists’ perspectives on AI applications range from outright enthusiasm to curiosity, skepticism, and fear [31]. These differences in opinion across radiologists were also visible for BoneXpert. Interestingly, none of the interviewees expressed fear of being replaced by AI. Rather, interviewees mentioned the need to reframe their professional identity and responsibilities as a consequence of the arrival of AI applications. For example, they envision radiologists of the future as “imaging consultants” who play an active role in an interdisciplinary patient-focused hospital environment [28]. An important element in this reframing process is creating the “right” narrative around AI. To overcome resistance by radiologists, developers and hospital management are framing AI applications as “co-pilots” enabling radiologists to perform better while staying in control.

Achieving acceptance of the referring clinicians is important since they are the potential final “customers” of the AI applications’ output. Interestingly, in three hospitals, we found that the referring clinicians did not trust the output of the AI application and redid a manual bone age analysis for every scan. Thus, just like the radiologists, the referring clinicians showed varying levels of acceptance of AI applications.

Finally, lacking jurisprudence from the European General Data Protection Regulation and the new Medical Device Regulation (MDR), which will come into effect in May 2020, leads to several *regulatory and legal uncertainties* for AI applications in radiology [32, 33]. Currently, CE marks are granted without requiring proof of the performance and added benefit for clinical practice. The new MDR requires CE certification through a notified body and necessitates a large increase in requirements on quality, safety, and post-market surveillance. Additionally, our interviewees expressed concerns about the unresolved question of legal responsibility for damage occurred due to e.g. false negatives and false positives resulting from an AI-generated diagnosis [34].

**Fig. 1:**
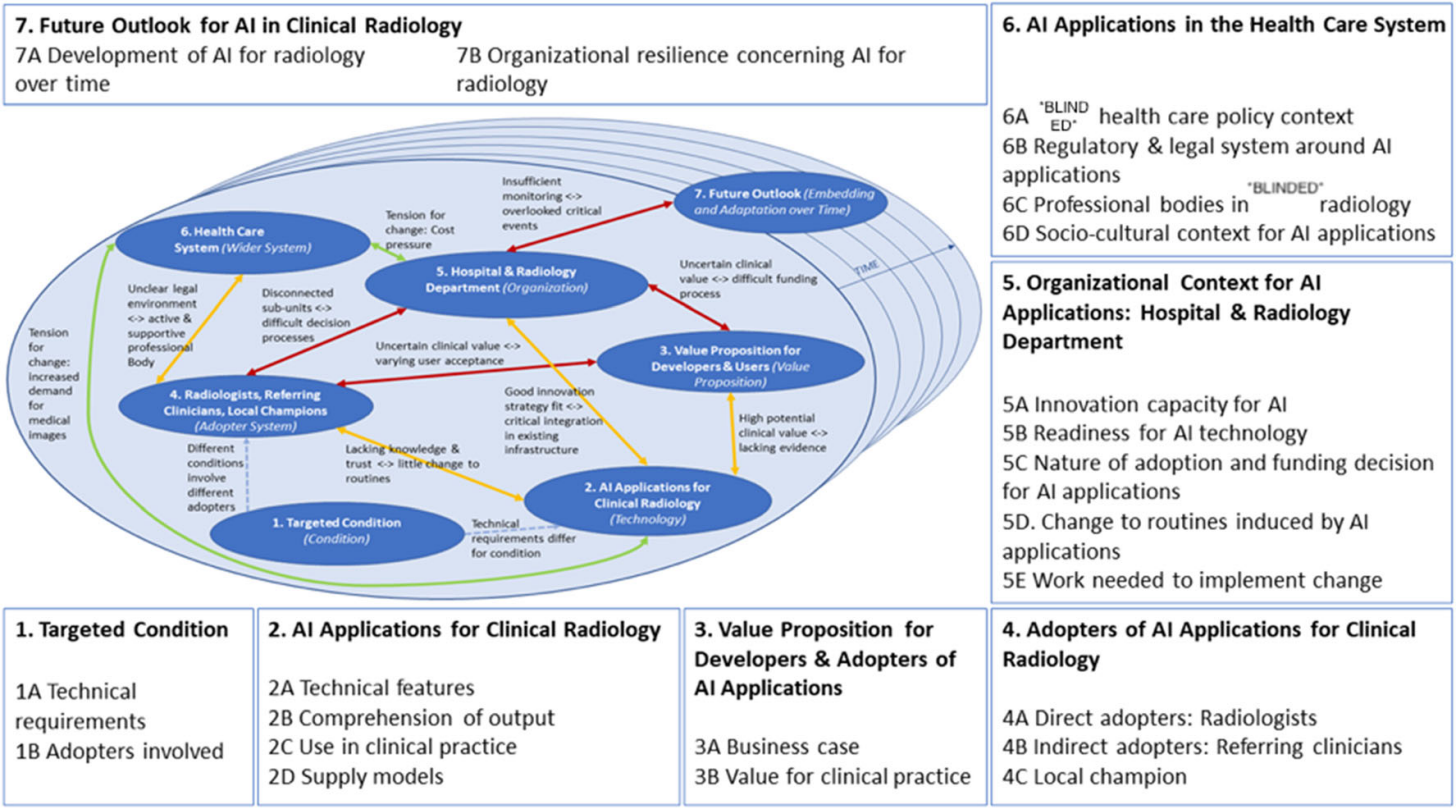
The NASSS framework [20], specified for AI applications in clinical radiology in The Netherlands

Based on the empirical findings, the NASSS framework was adapted and refined for the case of the implementation of AI applications in radiology. The main adaptation of the original NASSS model to form the NASSS for AI in radiology concerns the “adopter system” (Fig. 1). It is elaborated with local champions, radiologists as direct adopters of the technology, and referring clinicians as indirect adopters.

## Discussion

This research contributes to the existing empirical evidence on the implementation challenges of AI-based medical technologies. We identified lacking acceptance as one of the most important causes for non-adoption, abandonment, and thus a barrier to the successful implementation of AI applications in radiology. The determinants of radiologists’ acceptance of AI application found in this study are in line with evidence from surveys among radiologists [2, 35] and radiology residents [31] and earlier evidence on the determinants of clinicians’ acceptance of computerized decision support systems (CDSS): insufficient knowledge [5, 6, 36], trust [36, 37], change in clinician’s professional identity, and professional autonomy [36, 38]. We found local champions to play a crucial role in overcoming lacking acceptance of technology users. The significance of having a local champion had previously appeared in research on the adoption of telehealth systems [39], as well as on the implementation of CDSS [36]. Notably, a recent study on the implementation of CDSS in US American radiology departments also identified local champions as an important facilitator for implementation [40]. Both studies mention the local champions’ facilitating role in starting and advancing the implementation processes of CDSS.

Another implementation challenge we found, the role of evidence on innovation implementation, has been discussed extensively in the field of evidence-based healthcare [41]. Scientific evidence is an important determinant of innovation implementation for practitioners, a finding that also appears to hold for AI in radiology [8, 21, 41, 42]. It thus follows that AI applications for radiology reflect a trend in the field of medical imaging to engage with technologies that have yet to prove their promises of contributing to the improvement of the quality or efficiency of healthcare [43].

AI applications in radiology are predicted to not only support but also potentially automate certain medical decision processes, thereby calling into question the jobs of highly educated individuals. This element of job displacement due to automatization adds to the complexity of adoption and implementation processes in the field of health digitization. While the possibility of AI replacing radiologists, and thereby threatening their professional identity, was extensively mentioned in many recent radiology publications [5, 9, 16, 44], none of the interviewees in this research identified with this threat. This aligns with recent opinion surveys conducted among radiologists [2, 31, 35]. Across the healthcare field, radiologists already have the most digitized work environment [44] and they self-identify as logical frontrunners for using digitized supporting tools in their daily practice. In order to take on a leading role in the implementation of AI applications within the hospital, radiologists need to acquire AI literacy through complementary training [5–7].

Due to its exploratory nature and qualitative empirical approach, several limitations of the research need to be taken into consideration. This research only focuses on AI applications in Dutch radiology departments and could not be generalized to other healthcare systems. Across the cases, individuals with different roles and positions were interviewed, limiting the generalizability of the results to other hospitals in The Netherlands and beyond. Interviewees varied with regard to their related experience and knowledge, due to the early stage of implementation of AI in radiology practice. Therefore, the sample of interviewees is possibly biased towards individuals with a particular interest and above-average positive attitude towards AI applications.

In order to achieve better generalizability of the results, further research should investigate applications that present higher technical complexity than BoneXpert and represent a larger part of the diagnostic work done by radiologists. Furthermore, it is important to understand how country-specific political and social contexts determine the implementation processes. Future studies can identify specific technical challenges for the implementation of AI applications, e.g., datasets and associated requirements (their size, algorithms, and data heterogeneity). Additionally, future research should focus on the impact of the implementation of AI applications on the knowledge development of radiologists.

## Conclusion

Considering the great attention AI applications are receiving in radiology and other medical disciplines like pathology, understanding the barriers of and facilitators for the implementation of AI is important. One of the important facilitating factors is the presence of a “local champion,” an individual with a strong personal interest in AI applications who most often initiates and actively advances AI implementation in the organization. Among the most prominent hindering factors is the uncertain added value for clinical practice, which causes low acceptance of AI applications among adopters and complicates the mobilization of funds to acquire AI applications. Furthermore, the failure to include all relevant stakeholders in the planning, execution, and monitoring phase of the implementation of AI applications was found to be a major hindering factor. To increase the acceptance among adopters, more evidence of the added benefit of their AI applications in the clinical setting is needed. Also, all involved stakeholders (most notably radiologists and referring clinicians) should be included in the decisions for and the design of implementation processes of AI applications.

## Electronic supplementary material

ESM 1 (DOCX 24 kb)

## Abbreviations

AI: Artificial intelligence
CAD: Computerized aided diagnosis
CDSS: Computerized decision support systems
MDR: Medical Device Regulation
NASSS: Non-adoption, Abandonment, Scale-up, Spread, and Sustainability
NVvR: Radiological Society of The Netherlands
PACS: Picture archiving and communication systems

## Footnotes

1 BoneXpert is currently used in over 70 European hospitals, of which eight are located in The Netherlands [26]. From the eight hospitals, seven were included in the sample, due to non-response of the eighth hospital.

## References

[1] Obermeyer Z, Emanuel EJ (2016). Predicting the future — big data, machine learning, and clinical medicine. N Engl J Med.

[2] European Society of Radiology (ESR) (2019) Impact of artificial intelligence on radiology: a EuroAIM survey among members of the European Society of Radiology (ESR). Insights Imaging 10(1). 10.1186/s13244-019-0798-3 10.1186/s13244-019-0798-3 PMC6823335 31673823

[3] European Society of Radiology (ESR) (2019) What the radiologist should know about artificial intelligence – an ESR white paper. Insights Imaging 10(1):44. 10.1186/s13244-019-0738-2 10.1186/s13244-019-0738-2 PMC6449411 30949865

[4] Liew C (2018) The future of radiology augmented with Artificial Intelligence: a strategy for success. Eur J Radiol 102:152–156. 10.1016/j.ejrad.2018.03.019 10.1016/j.ejrad.2018.03.019 29685530

[5] Sogani J, Allen B Jr, Dreyer K, McGinty G (2019) Artificial intelligence in radiology: the ecosystem essential to improving patient care. Clin Imaging 59(July 2019):8–11. 10.1016/j.clinimag.2019.08.001 10.1016/j.clinimag.2019.08.001 31481284

[6] He J, Baxter SL, Xu J, Xu J, Zhou X, Zhang K (2019). The practical implementation of artificial intelligence technologies in medicine. Nat Med.

[7] Jha S, Topol EJ (2016). Adapting to artificial intelligence: Radiologists and pathologists as information specialists. JAMA.

[8] Char DS, Shah NH, Magnus D (2018). Implementing machine learning in health care ’ addressing ethical challenges. N Engl J Med.

[9] Chockley K, Emanuel E (2016). The end of radiology? Three threats to the future practice of radiology. J Am Coll Radiol.

[10] Choy G, Samir AE, Brink JA (2018). Current applications and future impact of machine learning in radiology. Radiology.

[11] Mazurowski MA (2019). Artificial intelligence may cause a significant disruption to the radiology workforce. J Am Coll Radiol.

[12] Dreyer KJ, Geis JR (2017). When machines think: Radiology’s next frontier. Radiology..

[13] Yu KH, Kohane IS (2019). Framing the challenges of artificial intelligence in medicine. BMJ Qual Saf.

[14] van Ginneken B, Schaefer-Prokop CM, Prokop M (2011). Computer-aided diagnosis: how to move from the laboratory to the clinic. Radiology..

[15] Kohli A, Jha S (2018). Why CAD failed in mammography. J Am Coll Radiol.

[16] Hosny A, Parmar C, Quackenbush J, Schwartz LH, Aerts HJWL (2018). Artificial intelligence in radiology. Nat Rev Cancer.

[17] Nishikawa RM, Bae KT (2018). Importance of better human-computer interaction in the era of deep learning: mammography computer-aided diagnosis as a use case. J Am Coll Radiol.

[18] Pope C, Halford S, Turnbull J, Prichard J, Calestani M, May C (2013) Using computer decision support systems in NHS emergency and urgent care: Ethnographic study using normalisation process theory. BMC Health Serv Res 13(1) 10.1186/1472-6963-13-111 PMC3614561 23522021

[19] Greenhalgh T, Robert G, Macfarlane F, Bate P, Kyriakidou O (2004). Diffusion of innovations in service organizations: systematic review and recommendations. Milbank Q.

[20] Greenhalgh T, Wherton J, Papoutsi C (2017). Beyond adoption: a new framework for theorizing and evaluating nonadoption, abandonment, and challenges to the scale-up, spread, and sustainability of health and care technologies. J Med Internet Res.

[21] Recht MP, Dewey M, Dreyer K et al (2020) Integrating artificial intelligence into the clinical practice of radiology: challenges and recommendations. Eur Radiol:1–9. 10.1007/s00330-020-06672-5 10.1007/s00330-020-06672-5 32064565

[22] AINED (2018) AI Voor Nederland. https://www.vno-ncw.nl/sites/default/files/aivnl_20181106_0.pdf. Accessed 2 Nov 2019

[23] WRR (2016) Big Data in Een Vrije En Veilige Samenleving. Vol 95. Den Haag. https://www.wrr.nl/publicaties/rapporten/2016/04/28/big-data-in-een-vrije-en-veilige-samenleving. Accessed 27 Oct 2019

[24] Nederland Digitaal (2019) Resultaten en opbrengsten van de Conferentie Nederland Digitaal 2019. In: Conferentie Nederland Digitaal. https://www.nederlanddigitaal.nl/documenten/publicaties/2019/03/21/opbrengsten-conferentie-nederland-digitaal-2019. Accessed 21 Nov 2019

[25] Lee H, Tajmir S, Lee J (2017). Fully automated deep learning system for bone age assessment. J Digit Imaging.

[26] Visiana (2018) Testimonials and references. https://www.bonexpert.com/documentation/testimonials-and-references. Accessed November 26, 2018.

[27] Ministerie van Volksgezondheid Welzijn en Sport (2018) Kamerbrief over Aanbieding Bestuurlijk Akkoord (Hoofdlijnenakkoord) Medisch-Specialistische Zorg 2019-2022. https://www.rijksoverheid.nl/documenten/kamerstukken/2018/06/04/kamerbrief-over-hoofdlijnenakkoord-medisch-specialistische-zorg-2019-2022. Accessed 21 Nov 2019

[28] Nederlandse Vereniging voor Radiologie (2016) De Rol van de Radioloog in 2020. Utrecht. https://www.radiologen.nl/system/files/bestanden/documenten/de_rol_van_de_radioloog_in_2020.pdf. Accessed 17 Nov 2019

[29] Nederlandse Vereniging van Ziekenhuizen (2018) Ziekenhuiszorg in Cijfers 2018. Utrecht. https://ziekenhuiszorgincijfers.nl/assets/uploads/NVZ-Brancherapport-2018.pdf

[30] Rubin DL (2019). Artificial intelligence in imaging: the radiologist’s role. J Am Coll Radiol.

[31] Pinto dos Santos D, Giese D, Brodehl S (2019). Medical students’ attitude towards artificial intelligence: a multicentre survey. Eur Radiol.

[32] Parikh RB, Obermeyer Z, Navathe AS (2019). Regulation of predictive analytics in medicine. Science.

[33] Tsang L, Kracov DA, Mulryne J (2017). The impact of artificial intelligence on medical innovation in the European Union and United States. Intellect Prop Technol Law J.

[34] Neri E, Coppola F, Miele V, Bibbolino C, Grassi R (2020) Artificial intelligence: who is responsible for the diagnosis? Radiol Med 0123456789. 10.1007/s11547-020-01135-9 10.1007/s11547-020-01135-9 32006241

[35] Waymel Q, Badr S, Demondion X, Cotten A, Jacques T (2019). Impact of the rise of artificial intelligence in radiology: what do radiologists think?. Diagn Interv Imaging.

[36] Liberati EG, Ruggiero F, Galuppo L (2017). What hinders the uptake of computerized decision support systems in hospitals? A qualitative study and framework for implementation. Implement Sci.

[37] Lugtenberg M, Weenink JW, Van Der Weijden T, Westert GP, Kool RB (2015). Implementation of multiple-domain covering computerized decision support systems in primary care: a focus group study on perceived barriers. BMC Med Inform Decis Mak.

[38] Bezemer T, de Groot MC, Blasse E (2019). A Human(e) factor in clinical decision support systems. J Med Internet Res.

[39] Wade V, Eliott J (2012). The role of the champion in telehealth service development: a qualitative analysis. J Telemed Telecare.

[40] Marcial LH, Johnston DS, Shapiro MR, Jacobs SR, Blumenfeld B, Rojas SL (2019). A qualitative framework-based evaluation of radiology clinical decision support initiatives: eliciting key factors to physician adoption in implementation. JAMIA Open.

[41] Turner S, D’Lima D, Hudson E (2017). Evidence use in decision-making on introducing innovations: a systematic scoping review with stakeholder feedback. Implement Sci.

[42] Urquhart R, Kendell C, Geldenhuys L (2019). The role of scientific evidence in decisions to adopt complex innovations in cancer care settings: A multiple case study in Nova Scotia, Canada. Implement Sci.

[43] Rai R, Kumar S, Batumalai V (2017). The integration of MRI in radiation therapy: collaboration of radiographers and radiation therapists. J Med Radiat Sci.

[44] Nawrocki T, Maldjian PD, Slasky SE, Contractor SG (2018). Artificial intelligence and radiology: have rumors of the radiologist’s demise been greatly exaggerated?. Acad Radiol.

