# Supplementary material for: Implementation of artificial intelligence (AI) applications in radiology: hindering and facilitating factors
Source: Eur Radiol. 2020 May 26;30(10):5525–32. doi: 10.1007/s00330-020-06946-y (PMC7476917; doi:10.1007/s00330-020-06946-y)
Supplement: Supplementary file 1 — (DOCX 24 kb) [file 330_2020_6946_MOESM1_ESM.docx]

Appendix A: Overview Affiliation and Field of Expertise Interviewees

| Interviewee ID | Role | Seniority | Case Affiliation | Additional Roles | Communication channel | Duration (min) |
| --- | --- | --- | --- | --- | --- | --- |
| IV1 | Radiologist | Senior | TKZ1 | Research collaboration with academic hospital | In Person | 75 |
| IV2 | Radiologist | Senior | TKZ2 | Educational Committee Professional Society | In Person | 43 |
| IV3 | Board of Directors | Senior | Professional Organization | Clinical radiologist/ PhD AI for Medical Imaging | In Person | 60 |
| IV4 | Data Scientist | Professor | UMC2 | Founder of several companies in AI for medical imaging | In Person | 45 |
| IV5 | Radiologist | Senior | UMC3 | Medical manager of pediatric radiology department + Responsible for residents training | In Person | 48 |
| IV6 | Radiologist | Senior | UMC3 | Medical Manager of Radiology Department, Research on AI in medical imaging | In Person | 45 |
| IV7 | Radiologist | Resident | UMC2 | PostDoc on AI in medical imaging | In Person | 22 |
| IV8 | Radiologist | Senior | TKZ2 | Medical Manager of Radiology Department | In Person | 50 |
| IV9 | Radiologist | Senior | AZ1 | Medical manager of radiology department | Telephone | 37 |
| IV10 | Radiologist | Senior | UMC1 |  | In Person | 27 |
| IV11 | Radiologist | Resident | UMC2 | PhD on AI in medical imaging | In Person | 25 |
| IV12 | Technician | Resident | TKZ2 |  | In Person | 48 |
| IV13 | Radiologist | Senior | UMC1 | Research in AI in medical imaging | In Person | 25 |
| IV14 | Clinical Physicist | Senior | TKZ1 |  | In Person | 50 |
| IV15 | Legal Advisor | Senior | TKZ1 |  | In Person | 54 |
| IV16 | Operational Manager Radiology Department | Senior | TKZ1 |  | In Person | 53 |
| IV17 | Innovation Manager | Junior | TKZ2 |  | In Person | 52 |
| IV18 | Innovation Manager | Senior | UMC1 |  | Telephone | 40 |
| IV19 | Advisor (Specialist for implementation of clinical interventions) | Senior | Professional Organization |  | In Person | 62 |
| IV20 | Innovation Manager | Senior | Innovation Technology Provider |  | In Person | 70 |
| IV21 | Radiologist | Senior | UMC4 | Research on AI in medical imaging | In Person | 72 |
| IV22 | Radiologist | Senior | UMC1 | Board member of Screening Organization | In Person | 62 |
| IV23 | Member of Management |  | Professional Organization |  | Telephone | 35 |
| IV24 | Data Scientist | Senior | UMC3 | Entrepreneur in AI for medical imaging | Telephone | 20 |
